# Influence of Kinesitherapy on Gait in Patients with Ischemic Stroke in the Chronic Period

**DOI:** 10.3889/oamjms.2015.107

**Published:** 2015-10-07

**Authors:** Danche Vasileva, Daniela Lubenova, Marija Mihova, Antoaneta Dimitrova, Kristin Grigorova-Petrova

**Affiliations:** 1 *University “Goce Delchev”, Faculty of Medical Sciences, Shtip, Republic of Macedonia*; 2 *National Sports Academy, Physical Therapy and Rehabilitation, Sofia, Bulgaria*; 3 *Ss Cyril and Methodius University of Skopje, Faculty of Computer Sciences and Engineering, Skopje, Republic of Macedonia*

**Keywords:** Kinesitherapy, Gait, Ischemic stroke, Chronic period, Neurodevelopment treatment, Physical activity

## Abstract

**AIM:**

The study aims to trace the influence of specialized kinesitherapeutic methodology (SKTM) on gait in patients with ischemic stroke in the chronic period (ISChP).

**MATERIAL AND METHODS:**

The study was conducted with 56 patients with ISChP (duration of the disease up to 1 year). For determining changes in gait before and after the treatment a cadence of gait and maximum movement speed were taken into consideration. To determine the cadence, steps are counted for covering 6 meters and 10 meters respectively. The maximum speed of the gait is determined in m / min by dividing undergone distance (m) and time (min).

**RESULTS:**

Patients were found to significantly normalize the parameters of gait. Compared to the initial data, there is a significant reduction in the number of steps on 6 and 10 meters and a tendency to increase the speed of gait, with the significant change during the 1st month with a level of significance of p <0.001.

**CONCLUSION:**

The applied specialized kinesitherapeutic methodology continued later as exercise program at home, which significantly improved gait cadence and speed of movement in patients with ischemic stroke in the chronic period and is with a supportive prolonged exposure.

## Introduction

Stroke is a major health problem and a leading cause of disability. Only 12% of patients are independent of basic daily activities at the end of the first week. In the long run, 25-74% of them have to rely on assistance from another person for basic daily activities such as eating, personal care and mobility [1, 2].

Despite the continuous development of neurorehabilitation many patients who suffered stroke have permanent disabilities in walking that affect their quality of life and ability to participate in daily activities [3, 4]. The central mechanisms for functional recovery are partly clarified. Undergoing processes that occur in the early period of brain injury are restitution, adaptive reorganization and compensatory strategies. In the late period (after 6 months) - organization of a new neural network covers the damaged premorbid network [5]. Recovery depends on the severity, location, extent of damage to the brain tissue and the limitation of the disease and neurorehabilitation [6].

Difficulties in walking in patients who suffered stroke are due to many factors, such as: reduced muscle strength, imbalance in the distribution of weight, impaired proprioception, increased Tendon reflexes, spasticity and infringement motor control [7]. Spastic increased muscle tone of the extensors on pareticc leg and contractures limit its flexion. During the swing phase it relatively extends, which compensates by lifting the pelvis and outsourcing paretic leg side resembling mowing. Electromyographic studies show bilateral changes in motor control - on the side of paresis and contralateral. Reciprocity of normal muscle contraction is replaced by co-activation. Alternative motor control is established by neuroimaging methods which is associated with the mechanisms of recovery of motor disorders [8].

One of the leading functional limitations that are a result of the stroke is significant gait deceleration. Therefore, the speed of the gait is the product of the length of the steps and rhythm, and reducing one of these parameters can lead to delay [9]. There is difficulty in overcoming obstacles. After stroke patients demonstrate significantly reduced success rate of overcoming obstacles, particularly if given limited time. They show normal strategies to overcome, but have delayed and reduced muscle reactions [10].

Typical gait disturbances that occur after stroke for infringements in the middle cerebral artery with consecutive one-sided weakness and spasticity are: reduced knee flexion in swing phase and support phase, hyperextension of the knee (dynamic recurvatum) in the phase of support and excessive plantar flexion of the ankle (equines) in swing phase and/or the phase of support. Each of these abnormalities has a potential negative effect and increase the need for walking energy [6, 11, 12].

The purpose of the study was to trace the influence of specialized kinesitherapeutic methodology (SKTM) based on the principles of motor control, motor training and modern guidelines of neurodevelopmental treatment (NDT) on the gait in patients with ischemic stroke in the chronic period (ISChP).

## Material and Methods

### Methodology of the Study

The study was conducted with 56 patients with ISChP (32 men and 24 women, average age 63.2 ± 8.8 years, duration of the disease up to 1 year) between 2012 and 2015, within the framework of specialized office for physical therapy at the Facuity of Medical Studies at the University “Goce Delchev” - Shtip, Macedonia and Specialized Hospital for post treatment and rehabilitation - Pancharevo - Sofia, Bulgaria.

The clinical characteristics of the patients are given in Table 1. According to the stage of Brunnstrom the severity of paresis is medium of the upper and lower limb. Patients can perform the following active movements: lifting the arm to 90 degrees, initial extension in elbow, wrist and fingers. In the lower limb possible movements are the following: extension in the knee and the initial dorsal flexion in the ankle joint. Muscle tone is slightly increased, according to the scale of Ashworth [13].

**Table 1:** Clinical characteristics of contingent at the beginning of the study

Age	Weight	Height	Brunnstrom- upper limb	Brunnstrom - lower limb	Ashworth - upper limb	Ashworth – lower limb
X̄±S_D_	X̄±S_D_	X̄±S_D_	X̄±S_D_	X̄±S_D_	X̄±S_D_	X̄±S_D_
63.2±8.8	77.9±10.1	169.2±6.4	4.2±0.7	4.8±0.6	1.6±0.6	1.1±0.5

X̄±S_D_ – average value and standard deviation

Due to homogeneity in the study, patients were selected by the following criteria: not have severe respiratory insufficiency, cardiovascular insufficiency (third functional class), uncontrolled diabetes mellitus, cognitive and memory disorders, acute thrombophlebitis, severe decubital ulcer, severe orthopedic disorders impairing coordination and gait, ischemic heart disease, malignancies, severe progressive neurological disorders and to have given a written consent to participate in the study. All patients were able to move independently or with assistance and without serious problems in communication, with a pre-prescribed medication by neurologists, including antithrombotic and antihypertensive drugs.

For the assessment of the initial functional status of the patients, Brunnstrom test was used, whereas for measuring objectivity of muscle tone before treatment - the scale of Ashworth [13]. For determining changes in gait before and after the treatment a cadence of gait and maximum movement speed were taken into consideration. To determine the cadence, steps are counted for covering 6 meters and 10 meters respectively. The patient is invited to walk that distance at a typical speed he chooses [14]. The maximum speed of the gait is determined in m/min by dividing undergone distance (m) and time (min). For this purpose, the patient is instructed to go through that distance with the highest walking speed, avoiding running.

All indicators in the patients were evaluated four times - at the beginning of the study, on the 10^th^ day, after the 1^st^ month and after the 3^rd^ month of the kinesitherapy.

### Methods of kinesitherapy

All patients were treated with antithrombotic drug and antihypertensive drugs and through a specialized 10-day SKTM, which later continues to be performed as adapted exercise program at home for a period of three months [15]. It was developed based on neurostimulative therapy of Bobath (Neurodevelop-mental Treatment - NDT) and principles of motor control. It is applied daily of moderate exercise intensity and duration of about 40-50 minutes. In the introductory part, the exercises are aimed at preparing the body for the upcoming exercises, gradual adaptation of the cardiovascular system (chest and diaphragmatic breathing). The main part of the kinesitherapy includes exercises for the transition from the occipital lying to standing exercises for upper limb and control of the shoulder girdle, lower limb exercises and control of the body, pelvis and walking. The final part includes relaxation exercises to patients.

### Statistics

The obtained data were processed statistically using descriptive analysis, analysis of variance and alternative analyzes. Paired Samples Test is used to compare the parameters at the beginning, on 10^th^ day, on the 1^st^ and 3^rd^ month after the kinesitherapy. Correlation analysis Pearson was used and p-value less than 0.01 were considered statistically significant.

## Results

**Figure 1:**
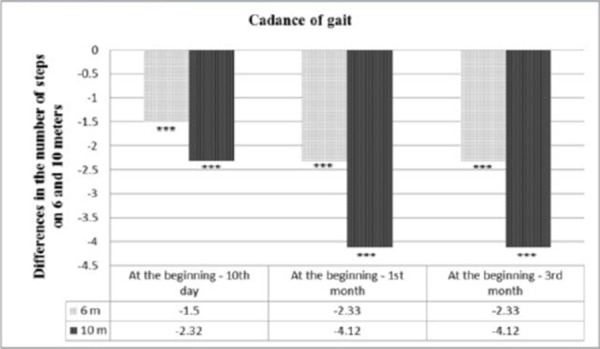
Changes in gait cadence of 6 m, 10 m, given the difference between the results and the beginning of the study, *** p <0.001 = significant difference compared to baseline.

**Figure 2:**
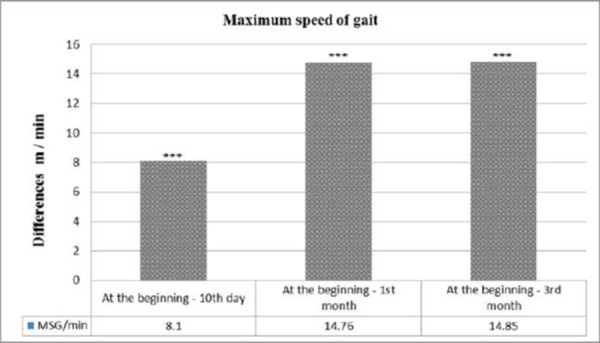
Changes in the speed of movement, given as the difference between the results obtained and baseline, *** p <0.001 = significant difference compared with baseline values.

The results of the study in patients with ISChP before treatment, on the 10^th^ day, 1^st^ month and 3^rd^ month after kinesitherapy are summarized in Table 2, and the ratio between obtained and baseline parameters studied and significance of changes in patients studied is presented in Figure 1 and 2.

**Table 2:** Prospective assessment on some characteristics of gait before and after kinesitherapy

Parameters	At the beginning (n=56)	10^th^ day (n=56)	1^st^ month (n=56)	3^rd^ month (n=56)
	X̄±S_D_	X̄±S_D_	X̄±S_D_	X̄±S_D_
6 meters (number of steps)	9.20±3.7	8.10±3.00 ***	7.20±2.30 ***	7.20±2.30 ***
10 meters (number of steps)	16.3±6.3	14.0±5.30 ***	12.2±4.10 ***	12.2±4.10***
Speed of gait (m / min)	31.3±16.6	39.4±16.6 ***	46.1±16.2 ***	46.1±15.2 ***

*X̄±S_D_ – average value and standard deviation*,

*** *p <0.001 = significant difference compared with values at the beginning of the study*.

Patients were found to substantially normalize the cadence of the gait of 6 and 10 meters walking. Compared to the initial data, there is a significant reduction in the number of steps (from 9.2 to 8.1 - for 6 meters and from 16.3 to 14.0 - for 10 meters walking distance) on the 10^th^ day of the observation. The first month after starting SKTM changes in gait cadence of 6 m and 10 m are expressed in reduced number of steps to 7.2 for 6 meters and 12.2 for 10 meters with a level of significance of p <0.001. Such are the changes in gait speed. The initial gait velocity is 31.3 m/min, which after the 10^th^ day tends to increase to 39.4 m/min and is with a biggest increase in the 1^st^ month (46.1 m/min), with a level of significance of p <0.001.

In the three functional parameters of the patients in the study, significant changes can be noticed up to 3^rd^ month, which changes are similar to the changes in the 1^st^ month, which means that SKTM has a long-term sustained impact.

Reducing the number of steps on 6m and 10m walking distance is associated with an increase in gait speed, like the change between the indicators register significant negative correlation (r = -0.851, p = 0.000 for 6 meters and r = -0.871 for 10 meters) the 10^th^ day that is significant to the 1^st^ month (r = -0.824, p = 0.000 for 6 meters and r = -0.814, p = 0.000 for 10 meters) and 3^rd^ month (r = -0.798, p = 0.000 for 6 meters and r = -0.796, p = 0.000 for 10 meters).

## Discussion

This study shows that functional limitations of gait in all patients with ISChP improved after applied 10-day SKTM that is continued as a program of exercises at home for 3 months. The number of steps decreases whereas the speed of gait increases to the monitored patients. In absolute terms, the improvement was most pronounced during the 1^st^ month of treatment. The effect is durable and lasts throughout the 3-month follow-up period.

These positive changes were associated with the included exercises of neurostimulative therapy of Bobath/Neurodevelopmental Treatment (NDT) for lower limb, control of body and pelvis that: normalize the control of the movements of the lower limb of the healthy and the affected side of the body, and consistency of motor response. They stimulate a response in the quadriceps femoris muscle and facilitate walking. Very important role plays the methodology of exercise in walking which leads to: normalizing the control of the body and upper limbs, improved dynamic control and facilitation of movement. Similar claims of other authors establish the superiority of NDT’s ability and speed of walking over other therapeutic approaches [16-18].

The beneficial effect in patients included in the study remained significant at longitudinal monitoring and is due to compliance with the basic principles of motor training, namely: active participation of the patient (if possible from the first hour), possibly more frequent application (which includes activities at home), focus (placed meaningful patient goals taking into account the usual activities carried out before the disease, movements similar to previous experience), motor activities with variations (repetition without repetition, performing motor activity in different versions, situations and conditions) [19]. The applied SKTM is oriented towards solving targeted functional activities by seeking active participation by the patient in order to gain experience and to seize the opportunities of the processes of neuroplasticity recovery. The patient should learn strategies to solve specific mobility problems, optimal orientation of the body, a good starting position, the ability to predict the sequence of movements and to enable the use of the skills that are adaptable [15].

The positive effect of the applied therapy is associated with stimulation of brain plasticity, using appropriate training in targeted tasks with high intensity when motivation is necessary [20].

The improvement was associated with a 3-month continuous application of the presented method. In patients with post-stroke hemiparesis, implementation of short 3-week neurorehabilitation improved kinetic parameters of gait, but did not affect the central programming step, which requires a reassessment of existing programs to include neurorehabilitation daily for at least 28 days of kinesitherapeutic program [21, 20]. For successful neurorehabilitation, optimal balance between duration of treatment and the patient’s clinical recovery is essential [5].

In conclusion, the applied specialized kinesitherapeutic methodology based on the principles of motor control, motor training and modern guidelines on neurostimulate therapy - Neurodevelopmental treatment (NDT) and continued later as exercise program at home, which significantly improves gait cadence and speed of movement in patients with ischemic stroke in the chronic period and is with a supportive prolonged exposure.
